# Mindfulness combined with cognitive motor dual task training in Parkinson’s disease with mild cognitive impairment

**DOI:** 10.1016/j.isci.2026.116197

**Published:** 2026-05-30

**Authors:** Rui Wang, Jing-Zhi Zhang, Si-Mao Xu, Jun Chen, Ben-xiang He

**Affiliations:** 1 Guilin Institute of Information Technology, Guilin, China; 2 Department of Rehabilitation Therapy, Nanjing Mingzhou Rehabilitation Hospital, Nanjing, China; 3 College of Physical Education and Health, Guangxi Normal University, Guilin, China; 4 Research institute of Sports Science, Guangxi Normal University, Guilin, China; 5 Sichuan Academy of Chinese Medicine Sciences, Chengdu, China

**Keywords:** Health sciences, Medicine, Medical specialty, Internal medicine, Neurology

## Abstract

Parkinson’s disease with mild cognitive impairment (PD-MCI) is a common non-motor manifestation that severely impairs functional independence and quality of life. A single-blind randomized controlled trial was conducted to evaluate whether mindfulness combined with cognitive-motor dual-task training (CMDT) improves multidimensional outcomes in patients with PD-MCI. Fifty three participants were randomly assigned to mindfulness plus CMDT, CMDT alone, or aerobic training for 4 weeks. Compared with the other groups, the combined intervention produced greater improvements in global cognitive function, motor symptoms, depression, sleep quality, quality of life, and postural stability. These findings support a multimodal rehabilitation strategy that simultaneously targets cognitive, motor, and neuropsychiatric pathways in PD-MCI.

## Highlights

• CMDT improved cognition and motor outcomes in PD-MCI patients
• Mindfulness further enhanced the effects of CMDT across outcomes
• Combined training improved depression, sleep, and quality of life
• Mindfulness plus CMDT produced the greatest postural stability gains

## Graphical abstract

**Figure:**
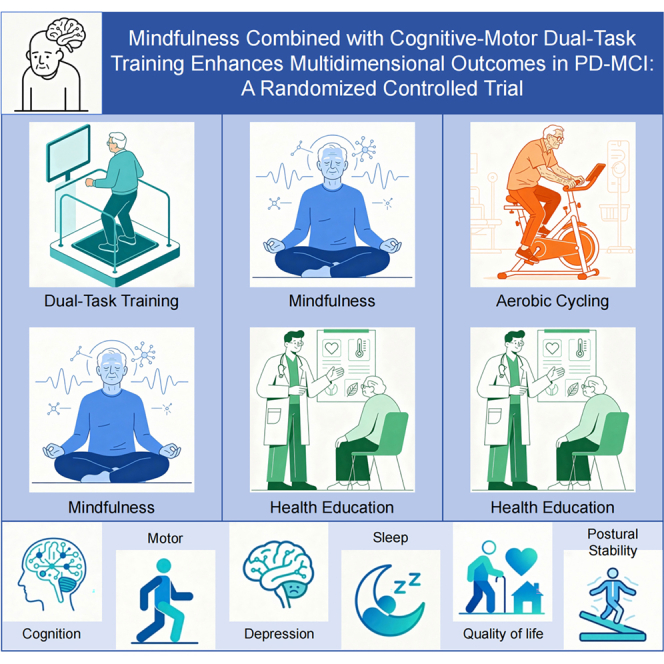

## Introduction

Parkinson’s disease (PD) is a common neurodegenerative disorder with a global prevalence of over 6 million.^1^ Motor symptoms are typical features of PD, such as tremor, rigidity, bradykinesia, and postural instability.^2^ However, increasing evidence indicates that health-related issues caused by non-motor symptoms are more significant.^3^ PD-mild cognitive impairment (PD-MCI) refers to the gradual decline in subjective or clinically confirmed cognitive abilities, accompanied by cognitive deficits confirmed on formal neuropsychological tests or global cognitive scales but without significant interference in functional independence.^4^ PD-MCI primarily affects memory, visuospatial, and executive functions.^5^ It is present in 25%–30% of patients with PD without dementia.^6^ Cognitive decline in PD-MCI patients may be difficult to detect but often accelerates later in the disease course, frequently accompanied by worsening motor symptoms.^7^ Most PD-MCI patients will progress to dementia over time, causing greater economic burden.^8^

The effectiveness of pharmacological interventions for PD-MCI remains limited, with no approved disease-specific therapies^9^ and inconsistent benefits reported across clinical trials.^10^^,^^11^^,^^12^^,^^13^^,^^14^^,^^15^^,^^16^ As a result, non-pharmacological interventions have received increasing attention. Despite the increasing application of cognitive training in PD,^6^ the certainty of evidence supporting pure cognitive training for cognitive improvement in PD-MCI remains low.^17^ Motor rehabilitation has emerged as a promising approach, not only for improving motor symptoms but also for exerting beneficial effects on cognitive function in PD. However, the overall cognitive effects of conventional motor interventions in PD-MCI remain modest.^7^^,^^18^ To address this limitation, Cognitive-motor dual-task training (CMDT), which integrates simultaneous or alternating cognitive and motor demands, has been proposed as a more targeted rehabilitation strategy.^19^ CMDT has been shown to improve both cognitive processing and motor performance in PD.^20^ Maidan et al. found that adding cognitive task components during exercise significantly altered the amplitude and lateralization of frontal lobe activation.^21^ At present, interactive CMDT based on game training system can further optimize engagement and training specificity, and is considered the most suitable for MCI.^22^^,^^23^ Liao et al. found that virtual reality-based interactive CMDT showed more improvements in divided attention and motor execution aspects in older adults with MCI than traditional CMDT.^24^

Mindfulness, a third wave cognitive-behavioral therapy developed based on meditation,^25^ which has been proven to improve cognitive function in the elderly and is equally effective in treating mental symptoms such as depression and anxiety.^26^^,^^27^ Mindfulness has been increasingly recommended as a supportive intervention for MCI^28^^,^^29^ but there is a lack of more rigorous research. A systematic review suggests that mindfulness should be considered as an adjunct with other therapies to further enhance its impact on psychological and cognitive outcomes of MCI.^30^

According to Huckans et al.’s theoretical rehabilitation model, MCI management should adopt multimodal rehabilitation strategies that focus on cognitive, motor, and neuropsychiatric symptoms.^31^ Mindfulness may strengthen the cognitive and psychological resources required for successful dual-task performance, while CMDT may reinforce the functional application of mindfulness-enhanced cognitive control in motor tasks. Combining these two modalities may produce synergistic effects in multiple impairment domains of PD-MCI.

Despite the theoretical possibility, no previous study has systematically evaluated the combined effects of mindfulness and CMDT in PD-MCI. Building on the multimodal rehabilitation model, this study used a balance training system interactive CMDT combined with mindfulness to intervene PD-MCI. We hypothesized that combining mindfulness with CMDT produces superior improvements in cognitive, motor, and neuropsychiatric symptoms compared with CMDT alone or aerobic training (AT).

### Methods

#### Design

This study was designed as a single-blind randomized controlled trial, following the CONSORT clinical trials reporting guidelines. The research process was conducted at Nanjing Mingzhou Rehabilitation Hospital and has obtained ethical approval from the hospital’s medical ethics management committee, with protocol number NJKF202503001. Registration completed on ClinicalTrials.gov (NCT06930742).

#### Participants

Participants were recruited from patients with PD admitted for clinical management between April and July 2025. Written informed consent was obtained from all participants in accordance with the Declaration of Helsinki.

Inclusion criteria were as follows: diagnosed of idiopathic PD according to the clinical diagnostic criteria of Movement Disorder Society (MDS)^32^; meeting MDS level 1 criteria for PD-MCI(4); Montreal Cognitive Assessment (MoCA) score < 26^33^; Mini-Mental State Examination (MMSE) score ≥ 24^34^; age ≥55 years; stable dopaminergic and/or cognition-related medication for ≥4 weeks prior before baseline and unchanged during the study.^35^

Exclusion criteria were as follows: current or past regular mindfulness practice; use of prescribed cognitive interventions or neuromodulation; history of acquired or traumatic brain injury; recent use of medications known to cause unstable or acute cognitive effects; illicit drug or alcohol abuse within the past 5 years; other neurological disorders affecting cognition; major psychiatric illness; severe visual, auditory, language, or motor impairments; any medical condition judged by investigators to substantially interfere with cognition, safety, or study adherence.

#### Interventions

Participants were randomly allocated to one of three groups: (1) mindfulness + CMDT (MC group); (2) health education + CMDT (CMDT group); (3) health education + AT (AT group). Each group received five 60 min intervention sessions per week for 4 weeks. Each session consisted of two 30 min components. Total intervention time, session frequency, and overall duration were matched across all groups. All participants received usual care during hospitalization, including medication management and support services but no specific structured cognitive or motor rehabilitation programs.^17^

The MC group received 30 min of mindfulness practice followed by 30 min of CMDT. Mindfulness was guided by an experienced therapist following established mindfulness-based intervention principles.^26^ Formal practices were conducted in a quiet room, including “body-scan,” “breath-focused,” and “loving-kindness” meditation. Participants were guided to sustain non-judgmental and receptive awareness toward bodily sensations, breathing rhythms, wandering thoughts, and emotional states, cultivating attentional stability and emotional regulation.^36^ Participants seated comfortably and therapist verbally guided them to observed internal experiences without avoidance or overinvestment. The sessions were followed in a structured progression: week 1, foundational diaphragmatic breathing, and guidance on posture attention and bodily sensations; week 2–3: focused-attention meditation on breath and cognitive-motor defusion strategies; week 4: open monitoring meditation, emotional regulation, and application of mindfulness during functional tasks. Each session included short informal exercises for inquiry and reflection, to reinforce attentional control and self-regulation and extend them to daily activities.

CMDT was delivered using EasyTech Libra proprioceptive balance training system (EasyTech, Italy), an electronic balance platform designed for proprioceptive and neuromuscular training. The system consists of a 42 × 42 cm balance board equipped with high-precision pressure sensors that continuously capture real-time center-of-pressure (COP) data and provide visual and auditory biofeedback by an external display interface. The device allows adjustable levels of instability, enabling progressive modulation of task difficulty. By integrating unstable surface training with real-time biofeedback, the system simultaneously challenges postural control and cognitive processing. Participants performed game-based balance tasks by shifting their body weight on the balance board. Multiple gamified modules covering balance, postural control, attentional shifting, visuospatial processing, working memory, and executive function. Representative games included: skiing, controlling a sled to avoid obstacles by leaning left or right; pinball, constantly intercepting bouncing balls, challenging anticipatory postural adjustments; balloon-poking, rapid weight-shift responses to strike approaching targets; and arithmetic or sequencing games requiring simultaneous balance control cognitive processing. Each session consisted of five diverse games, allowing coverage of different motor-cognitive domains. Short breaks were interspersed between games to prevent fatigue or habituation. Task difficulty was progressively adjusted over the 4-week intervention period. The first week emphasized static maintenance with simple cognitive demands; add low-amplitude weight shifts in the second week; weeks 3–4 included larger excursion ranges, faster movement requirements, narrower stability margins, reduced reaction time windows, and increasingly complex cognitive loads.

The CMDT group received 30 min of health education followed by 30 min of CMDT. The CMDT session was identical to the MC group. Health education served as an attention placebo control, adapted from the Stanford Chronic Disease Self-Management Program.^37^ The content included general topics such as medication safety, nutrition, sleep hygiene, stress awareness, and daily self-management.^38^ The sessions matched the mindfulness intervention in format, duration, and venue but did not include any mindfulness or structured cognitive training components.

The AT group received 30 min of health education followed by 30 min of AT. The health education session was identical to the CMDT group. AT sessions were conducted on a cycle ergometer at a moderate intensity level (approximately 40%–60% heart rate reserve). A therapist provided supervision to ensure safety and adherence. The sessions included warm-up, continuous cycling, and short breaks.

#### Outcome measures

Outcome measures were assessed at baseline (prior to randomization) and within 24 h after completion of the 4-week intervention period. A comprehensive battery of clinical, instrument, and patient-reported outcomes was employed to assess the multidimensional effects. Outcome selection was guided by the core diagnostic domains of PD-MCI,^1^ the hypothesized mechanisms of mindfulness and CMDT,^2^ and recommendations from the MDS for PD-related clinical trials.

The primary outcome is global cognitive function, assessed using the MoCA.^33^ It assesses eight cognitive domains including executive function, visual-spatial ability, language, memory, attention, abstraction, orientation, and naming. Scores range from 0 to 30, with lower scores indicating severe cognitive impairment.

Motor symptoms were assessed using the Unified Parkinson’s Disease Rating Scale-III (UPDRS-III).^39^ The scale quantifies bradykinesia, tremor, rigidity, gait, and postural stability. Total score ranges 0–132, with higher scores indicating more severe motor symptoms impairment. Measured in the “ON” drug-state to minimize variability.

Depressive symptoms were assessed using the Hamilton Depression Scale (HAMD), a structured clinical interview that has been widely validated in neurological populations and is sensitive to mild affective symptoms common in PD-MCI.^40^ The scale assesses emotional, cognitive, and somatic depressive features, including 24 items, with a total score range 0–96. The higher the score, the more severe are the depressive symptoms.

Sleep-related symptoms were assessed with PD Sleep Scale-2 (PDSS-2).^41^ The scale assesses three domains including insomnia, nocturnal motor symptoms, and non-motor symptoms. Total score ranges 0–60, with higher scores indicating more severe sleep disturbance.

Quality of life was assessed with PD Quality of Life Questionnaire (PDQ).^42^ The questionnaire assesses mobility, activity daily living, emotional well-being, stigma, social support, cognition, communication, and bodily discomfort, including 39 items, with a total score range 0–156. The higher the score, the poorer is perceived the quality of life.

Postural stability was assessed using built-in balance assessment module of the Libra proprioceptive balance training system. Participants stood on the balance board for 60 s. Following an audio cue, they were instructed to maintain a stable upright stance while minimizing excessive displacement of their COP. The board’s embedded sensors continuously captured COP trajectories at high sampling frequency, transmitting real-time data to the screen. Participants were asked to keep the COP trace as close as possible to the midline reference. The outcome of this assessment was the COP displacement area (COPDA), automatically calculated by the system based on the trajectory envelope formed during the 60 s testing period. Smaller COPDA indicate better postural stability.

#### Randomization and allocation

External personnel prepared numbered cards, placing them in sealed and opaque envelopes. After enrollment, each participant selected an envelope. A researcher not involved in assessment viewed the numbers and assigned participants to one of the three groups. Participants were instructed not to discuss their intervention with other participants or assessors. Outcome assessor and statistical analyst were blinded to both intervention and allocation.

#### Sample size determine

Sample size was calculated using G∗power 3.1.9.7 software based on a repeated measures analysis of variance (within-between interaction) design. The effect size “f” was calculated to be 0.45 based on a previous randomized controlled trial that reported significant group × time interaction effects on MoCA scores.^43^ To avoid overestimation and ensure a conservative calculation, a medium effect size (f = 0.25) was used in the final analysis. The calculation assumed three groups and two measurement time points. The significance level (α) was set at 0.05 and statistical power (1−β) at 0.80. The correlation among repeated measures was assumed to be 0.5, and the non-sphericity correction “ε” was set to 1. The required total sample size was calculated to be 42. Considering potential dropout, a total of 60 participants were recruited.

#### Statistical analysis

The Shapiro-Wilk test was used to examine the normality of quantitative variables. Since all variables followed a normal distribution, a two-way repeated-measures analysis of variance was performed to examine the effects of group, time, and their interaction on all outcome measures. The primary effect of interest was the group × time interaction. When a significant group × time interaction effect was identified, post hoc pairwise comparisons were conducted for within-group (pre vs. post) and between-group comparisons. The resulting *p* values were adjusted using the false discovery rate (FDR) correction based on the Benjamini-Hochberg procedure. Categorical variables (e.g., gender) were analyzed using chi-squared tests for baseline comparisons. An intention-to-treat (ITT) analysis was additionally performed as a sensitivity analysis using the last observation carried forward (LOCF) method to handle missing post-intervention data. The primary analysis was conducted based on the per-protocol population. All significance tests were two-tailed, with statistical significance set at *p* values < 0.05. Statistical analyses were conducted using SPSS 21.0 software.

## Results

### Baseline demographics and measures

**Figure 1:**
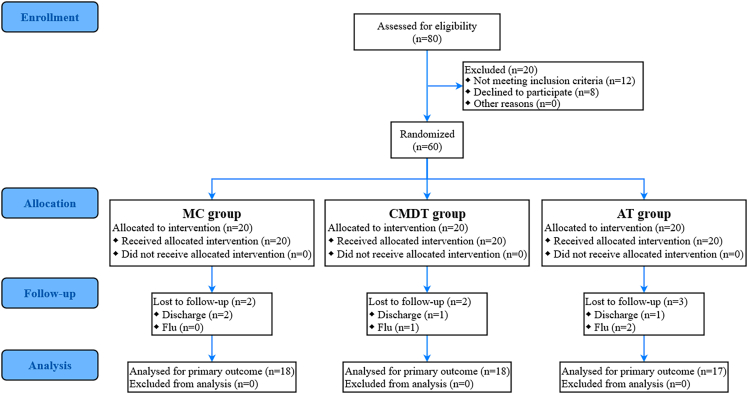
CONSORT flow diagram of participant enrollment, allocation, follow-up, and analysis MC, mindfulness + cognitive-motor dual-task training; CMDT, cognitive-motor dual-task training; AT, aerobic training.

A total of 80 participants were initially enrolled, of which 60 passed the screening. They were divided into the MC group, CMDT group, and AT group, with 20 participants in each group. Seven participants dropped out due to discharge or flu. Data from last 53 patients were analyzed. The participant flow is presented in accordance with CONSORT guidelines (Figure 1). No adverse events occurred during the intervention period. Table 1 presents no significant differences in baseline demographics and measures among the three groups (*p* > 0.05).

**Table 1:** Demographic and clinical characteristics of the patients at baseline

Characteristics	MC group (*n* = 18)<	CMDT group (*n* = 18)	AT group (*n* = 17)	*F* (2, 50)	*p*	*ηp*2
Gender, male/female, *n*	11/7	12/6	10/7	–	0.885	–
Age, years, mean (SD)	69.2 (4.6)	68.6 (5.2)	68.4 (6.3)	0.11	0.898	<0.01
MoCA, mean (SD)	22.1 (1.7)	22.1 (1.5)	21.9 (2.1)	0.04	0.961	<0.01
UPDRS-III, mean (SD)	26.6 (3.3)	25.6 (3.6)	25.9 (3.3)	0.40	0.673	0.02
HAMD, mean (SD)	11.5 (2.0)	11.3 (2.2)	11.9 (2.3)	0.29	0.747	0.01
PDSS-2, mean (SD)	13.3 (1.8)	12.8 (1.9)	13.5 (1.5)	0.84	0.437	0.03
PDQ, mean (SD)	37.2 (6.7)	36.1 (6.7)	37.8 (6.7)	0.29	0.747	0.01
COPDA, cm^2^, mean (SD)	322.7 (45.8)	340.7 (54.4)	329.4 (58.5)	0.53	0.591	0.02

MC, mindfulness + cognitive-motor dual-task training; CMDT, cognitive-motor dual-task training; AT, aerobic training; SD, standard deviation; MoCA, Montreal cognitive assessment; UPDRS-III, Unified Parkinson’s Disease Rating Scale-III; HAMD, Hamilton Depression Scale; PDSS-2, Parkinson’s disease Sleep Scale-2; PDQ, Parkinson’s disease Quality of Life Questionnaire; COPDA, center of pressure displacement area; cm^2^, square centimeter.

### Outcome measures

**Figure 2:**
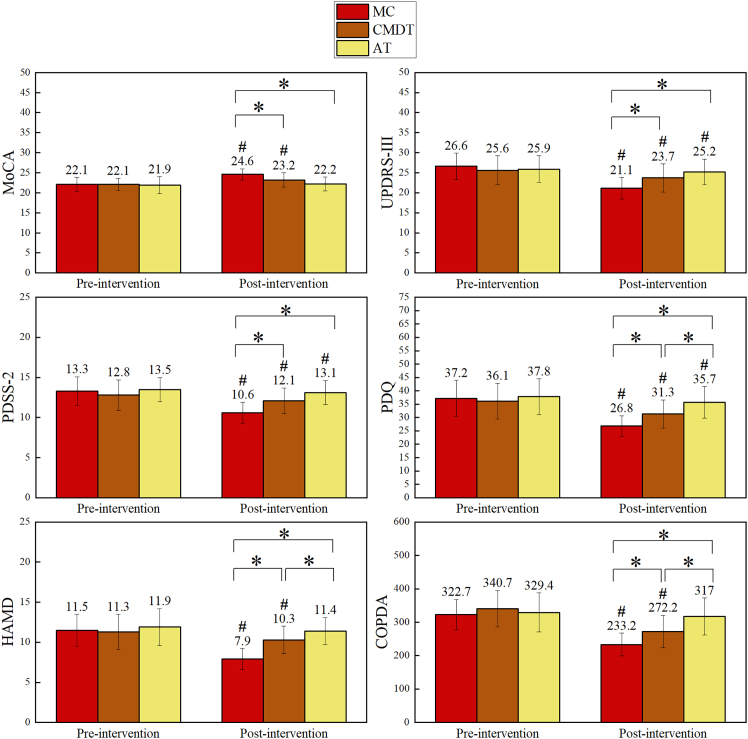
Changes in outcome measures across the three groups before and after the intervention The six images represent six outcome measures: MoCA, UPDRS-III, PDSS-2, PDQ, HAMD, and COPDA, respectively. Different colors indicate the three intervention groups: MC group (*n* = 18), CMDT group (*n* = 18), and AT group (*n* = 17). Values are presented as means, with error bars indicating standard deviations. Significant group × time interaction effects were observed for all outcomes (*p* < 0.05) based on two-way repeated-measures analysis of variance. #*p* < 0.05 for post hoc within-group pairwise comparisons (pre vs. post); ∗*p* < 0.05 for post hoc between-group pairwise comparisons at post-intervention following significant two-way repeated-measures analysis of variance. MC, mindfulness + cognitive-motor dual-task training; CMDT, cognitive-motor dual-task training; AT, aerobic training; MoCA, Montreal cognitive assessment; UPDRS-III, Unified Parkinson’s Disease Rating Scale-III; PDSS-2, Parkinson’s disease Sleep Scale-2; PDQ, Parkinson’s disease Quality of Life Questionnaire; HAMD, Hamilton Depression Scale; COPDA, center of pressure displacement area.

Significant group × time interactions were observed across all outcomes, with the MC group consistently demonstrating the greatest improvements. Sensitivity analyses using the ITT approach yielded comparable results across all measures. Table 2 and Figure 2 present the outcome measures across time for each group, and Table 3 presents the comparisons between groups based on significant group × time interaction effect.

**Table 2:** Within-group pre-post changes in outcome measures

Variables	Group	*F* (1, 50)<	*p*	*ηp*^2^	Mean difference	Std. Error	95% CI	
							Lower bound	Upper bound
**MoCA**	MC	212.03	<0.001	0.81	−2.50	0.17	−2.85	−2.16
	CMDT	46.18	<0.001	0.48	−1.17	0.17	−1.51	−0.82
	AT	2.77	0.102	0.05	−0.29	0.18	−0.65	0.06
**UPDRS-III**	MC	435.72	<0.001	0.90	5.56	0.27	5.02	6.09
	CMDT	50.37	<0.001	0.51	1.89	0.27	1.35	2.42
	AT	6.64	0.013	0.12	0.71	0.27	0.16	1.26
**HAMD**	MC	233.73	<0.001	0.82	3.56	0.23	3.09	4.02
	CMDT	18.49	<0.001	0.27	1.00	0.23	0.53	1.47
	AT	3.87	0.055	0.07	0.47	0.24	−0.01	0.95
**PDSS-2**	MC	376.19	<0.001	0.88	2.72	0.14	2.44	3.00
	CMDT	22.56	<0.001	0.31	0.67	0.14	0.39	0.95
	AT	8.13	0.006	0.14	0.41	0.14	0.12	0.70
**PDQ**	MC	347.49	<0.001	0.87	10.33	0.55	9.22	11.45
	CMDT	72.57	<0.001	0.59	4.72	0.55	3.61	5.84
	AT	13.03	0.001	0.21	2.06	0.57	0.91	3.21
**COPDA**	MC	219.75	<0.001	0.82	89.42	6.03	77.31	101.54
	CMDT	129.04	<0.001	0.72	68.52	6.03	56.41	80.64
	AT	3.99	0.051	0.07	12.40	6.21	−0.07	24.87

MoCA, Montreal cognitive assessment; UPDRS-III, Unified Parkinson’s Disease Rating Scale-III; HAMD, Hamilton Depression Scale; PDSS-2, Parkinson’s disease Sleep Scale-2; PDQ, Parkinson’s disease Quality of Life Questionnaire; COPDA, center of pressure displacement area; cm^2^, square centimeter; MC, mindfulness + cognitive-motor dual-task training; CMDT, cognitive-motor dual-task training; AT, aerobic training; CI, confidence interval.

**Table 3:** Comparisons in total outcome measures among the three groups post-intervention

Variables	Groups			*F* (2, 50)	*p*	*ηp*^2^	Pairwise comparison					
	MC mean (SD)	CMDT mean (SD)	AT mean (SD)				Groups	Mean difference	Std. Error	*p*	95% CI	
											Lower bound	Upper bound
**MoCA**	24.6 (1.4)	23.2 (1.8)	22.2 (1.7)	9.16	<0.001	0.27	MC-CMDT	1.39	0.55	0.023	0.28	2.50
							MC-AT	2.38	0.56	<0.001	1.25	3.50
							CMDT-AT	0.99	0.56	0.083	−0.14	2.11
**UPDRS-III**	21.1 (2.7)	23.7 (3.5)	25.2 (3.2)	7.98	0.001	0.24	MC-CMDT	−2.67	1.05	0.021	−4.77	0.56
							MC-AT	−4.18	1.06	<0.001	−6.31	−2.05
							CMDT-AT	−1.51	1.06	0.161	−3.65	0.62
**HAMD**	7.9 (1.3)	10.3 (1.7)	11.4 (1.7)	22.55	<0.001	0.47	MC-CMDT	−2.39	0.52	<0.001	−3.44	−1.34
							MC-AT	−3.47	0.53	<0.001	−4.53	−2.40
							CMDT-AT	−1.08	0.53	0.047	−2.14	0.01
**PDSS-2**	10.6 (1.3)	12.1 (1.6)	13.1 (1.5)	12.84	<0.001	0.34	MC-CMDT	−1.56	0.50	0.005	−2.57	−0.55
							MC-AT	−2.56	0.51	<0.001	−3.59	−1.54
							CMDT-AT	−1.01	0.51	0.054	−2.03	0.02
**PDQ**	26.8 (3.9)	31.3 (5.3)	35.7 (5.9)	13.16	<0.001	0.35	MC-CMDT	−4.50	1.71	0.017	−7.92	−1.08
							MC-AT	−8.87	1.73	<0.001	−12.35	−5.40
							CMDT-AT	−4.37	1.73	0.015	−7.85	−0.90
**COPDA (cm**^**2**^**)**	233.2 (34.2)	272.2 (49.1)	317.0 (55.7)	15.36	<0.001	0.38	MC-CMDT	−38.94	14.90	0.012	−68.87	−9.02
							MC-AT	−83.76	15.12	<0.001	−114.13	−53.40
							CMDT-AT	−44.82	15.12	0.008	−75.18	−14.45

MoCA, Montreal cognitive assessment; UPDRS-III, Unified Parkinson’s Disease Rating Scale-III; HAMD, Hamilton Depression Scale; PDSS-2, Parkinson’s disease Sleep Scale-2; PDQ, Parkinson’s disease Quality of Life Questionnaire; COPDA, center of pressure displacement area; cm^2^, square centimeter; MC, mindfulness + cognitive-motor dual-task training; CMDT, cognitive-motor dual-task training; AT, aerobic training; SD, standard deviation; CI, confidence interval.

For global cognitive function, a significant group × time interaction was observed for MoCA scores (F(2, 50) = 40.86, *p* < 0.001, ηp^2^ = 0.62). Post hoc analyses showed that both the MC and CMDT groups improved significantly after the intervention (*p* < 0.05), whereas no significant change was observed in the AT group (*p* > 0.05). The improvement in the MC group was significantly greater than that in both the CMDT and AT groups (*p* < 0.05), with no difference between the CMDT and AT groups (*p* > 0.05).

For motor outcomes, significant group × time interactions were observed for both UPDRS-III scores (F(2, 50) = 88.65, *p* < 0.001, ηp^2^ = 0.78) and COPDA (F(2, 50) = 42.07, *p* < 0.001, ηp^2^ = 0.63). UPDRS-III scores decreased significantly in all the three groups after the intervention (*p* < 0.05), whereas COPDA values decreased significantly in the MC and CMDT groups only (*p* < 0.05), with no significant change in the AT group (*p* > 0.05). For both measures, the MC group showed significantly greater improvement than the CMDT and AT groups (*p* < 0.05). In addition, the CMDT group demonstrated greater improvement than the AT group for COPDA (*p* < 0.05) but not for UPDRS-III (*p* > 0.05).

For non-motor outcomes, significant group × time interactions were observed for HAMD (F(2, 50) = 49.55, *p* < 0.001, ηp^2^ = 0.67), PDSS-2 (F(2, 50) = 80.41, *p* < 0.001, ηp^2^ = 0.76), and PDQ (F(2, 50) = 56.84, *p* < 0.001, ηp^2^ = 0.70). HAMD scores decreased significantly in the MC and CMDT groups (*p* < 0.05), whereas no significant change was observed in the AT group (*p* > 0.05). PDSS-2 and PDQ scores improved significantly in all three groups (*p* < 0.05). Across all non-motor outcomes, the MC group showed significantly greater improvements than the other groups (*p* < 0.05). The CMDT group also demonstrated greater improvements than the AT group for HAMD and PDQ (*p* < 0.05) but not for PDSS-2.

## Discussion

The present study evaluated the effects of mindfulness combined with CMDT, CMDT alone, and AT on cognitive, motor, depression, sleep, quality of life, and postural stability outcomes in PD-MCI. The MC group showed the most significant improvements. These findings supported the hypothesis that combining mindfulness with CMDT has multidimensional effects in PD-MCI rehabilitation.

CMDT, as a simultaneous cognitive-motor load, can enhance neural pathways that supporting task switching, working memory, and dual-task coordination, which are involved in the central domain impaired in PD-MCI.^44^ The simultaneous engagement of cognitive-motor processes also promotes increased cerebral blood flow and glucose metabolism in the dorsolateral prefrontal cortex and anterior cingulate cortex, which are crucial for attention allocation and executive control.^45^ A meta-analysis by Ali et al. showed a small-to-medium positive effect of CMDT on cognitive functions in older adults with cognitive impairment.^46^ Regarding global cognitive function, significant improvements were observed in the MC and CMDT groups. The superior effect of the MC group may be attributed to the additional effect of mindfulness. Mindfulness can enhance sustained attention, reduce cognitive interference from ruminative thoughts, and improved neural flexibility.^47^ Cognitive performance may benefit from reduced cognitive load and increased awareness of bodily cues cultivated through mindfulness. This pattern of mindfulness combined with CMDT corresponds to the neurocognitive features of PD-MCI, where deficits mainly involve attention, execution, and processing function.^48^ Mindfulness may enhance these functions by improving top-down cognitive control and inhibitory regulation through activation of the dorsolateral prefrontal cortex and anterior cingulate cortex.^49^ It also reduces cognitive interference related to rumination and anxiety.^50^ A study by Pickut et al. investigated brain changes after mindfulness in PD patients and found increased gray matter density in the hippocampus, amygdala, caudate nucleus, left thalamus, temporoparietal junction, cuneus, left occipital lobe, and left parahippocampal gyrus.^51^ Whitfield et al. found that mindfulness also emphasizes strengthening the mental skills, thereby translating to improved performance on objective cognition.^52^ In contrast, aerobic cycling performed in a seated and cognitively simple format lacks the cognitive stimulation needed to induce measurable changes in global cognition. The short-term effect of aerobic activity alone on overall cognition is relatively weak.^53^

Motor symptoms improved significantly in all groups. Reflecting that any format of structured physical activity can transiently improve motor performance in PD-MCI.^54^ The MC and CMDT groups achieved greater reductions than AT, indicating that cognitively enriched training may better target cognitive issues underlying motor symptoms. Scholars have proposed that there is a close interaction between motor and cognitive abilities, and improvements may reflect in more efficient allocation of limited attention resources.^55^ Dual-task challenges closely resemble real-life mobility demands in PD, such as walking while planning or reacting to external cues.^20^ In completing balance tasks, participants must properly mobilize cognitive processing to effectively use external information to maintain posture stability.^56^ CMDT-based interventions have been shown to improve motor adaptability and bradykinesia by strengthening cortical-basal ganglia-cerebellar coordination.^57^ The cognitive demands of dual-tasks activate the prefrontal-parietal network, enhancing neural efficiency through increased dendritic branching and synaptogenesis. Concurrent activation of cognitive and motor circuits during CMDT appears to strengthen connectivity between the cerebellum and prefrontal cortex, improving both motor learning and executive function.^58^ Shin et al. found that CMDT-based balance training improved balance and walking ability more than simple balance training.^59^ Evidence also suggests that adding cognitive tasks to balance training improves motor and cognitive abilities in patients with neurological disorders.^60^ Mindfulness not only enhances cognition but also improves postural awareness, reduces excessive muscular co-contraction, enhances sensorimotor integration, and suppresses abnormal movement to further improve athletic performance. Dissanayaka et al. observed improvements in postural instability, gait, and rigidity after mindfulness interventions in PD patients.^61^ Ewok et al. similarly reported significant improvements in UPDRS scores following mindfulness yoga.^62^ This may explain why the MC group demonstrated the greatest improvement in UPDRS-III scores.

The interaction between depression and cognitive impairment may lead to the occurrence and development of cognitive frailty.^63^ Depressive symptoms improved in the MC and CMDT groups. Ye et al. pointed out that CMDT can effectively improve depression in older adults with MCI.^64^ The cognitive engagement and motor activation of CMDT may promote release of neurotrophic factors, enhance synaptic plasticity, and strengthen motivational neural pathways, thereby alleviating depressive symptoms. The interactivity and gamified structure of CMDT can also improve perceived competence and self-efficacy, which are critical psychological factors influencing mood in PD.^65^ A meta-analysis suggests that mindfulness may alleviate depressive symptoms in elderly patients with MCI.^66^ In PD, dopaminergic depletion in the mesocorticolimbic system and serotonergic dysfunction contribute to mood dysregulation. Mindfulness may partially compensate for these deficits by increasing dopaminergic tone, improving reward processing, and normalizing stress-related hypothalamic-pituitary-adrenal axis activity.^67^ The greater improvement in the MC group compared to CMDT possibly reflects the synergistic effect of mindfulness provided emotional regulation and CMDT-induced neuroplasticity. Evidence indicates that mindfulness induced stress reduction is associated with changes in gray matter density in the amygdala and hippocampus.^67^ Studies based on mindfulness interventions have consistently shown increased activity and structural changes in the insula, anterior and posterior cingulate cortex, striatum, and medial and dorsolateral prefrontal cortex.^68^^,^^69^ These regions play key roles in attention control, emotion regulation, and self-awareness, and largely overlap with regions activated during acute stress. Mindfulness also enhances resting-state connectivity within the default mode network, reducing maladaptive rumination that commonly contributes to depressive symptoms in PD-MCI.

Motor activity promotes sleep by enhancing circadian rhythm stability and reducing nocturnal motor symptoms.^70^ Sleep quality improved in all three groups. CMDT provides additional cognitive engagement that may reduce daytime lethargy and improve mental alertness, indirectly benefiting night time sleep patterns. Nonetheless, greater improvements were observed in the MC group compared with the CMDT and AT groups. Black et al. found that mindfulness has clinical usefulness in treating moderate sleep problems and daytime impairment in older adults.^71^ Mindfulness directly targets two core contributors of sleep disturbance: cognitive hyperarousal and emotional reactivity. At the physiological level, mindfulness increases parasympathetic activity, reduces sympathetic tone, and downregulates hypothalamic-pituitary-adrenal axis activation, all of which support sleep onset and continuity.^67^ Mindfulness may also reduce pre-sleep worry and intrusive thoughts by engaging attentional networks and promoting non-reactive mental stance. By improving mood, reducing depression, and enhancing internal bodily awareness, the MC group may also have achieved improvements in nocturnal rest, which cannot be achieved solely through physical training.^72^

PD-MCI patients often experience daily functional decline related to cognition, motor, depression, and sleep^73^ as the PDQ integrates these symptom domains. Interventions simultaneously targeting multiple pathways have naturally resulted in greater overall improvement in quality of life. As discussed earlier, CMDT can play a certain role in the cognition, motor, depression, and sleep of PD-MCI, indirectly improving quality of life. Zhou et al. found that CMDT improved cognitive function and psychological status of elderly people with cognitive frailty and depression, thereby improving quality of life.^58^ CMDT may enhance functional mobility, posture control, and attentional capacity during daily activities. By simulating multitasking situations, CMDT can reduce fear of movement and enhance autonomy, both of which are closely associated with PDQ.^74^ Building on these effects, the greatest improvement in the MC group may be attributed to the complementary mechanisms of mindfulness. CMDT primarily enhances functional abilities for daily tasks, while mindfulness optimizes attentional stability and emotional resilience, potentially allowing patients to perform functional activities in daily life. Mindfulness can reduce maladaptive reactions to motor limitations, increase tolerance for cognitive lapses, and foster acceptance-based coping strategies to mitigate distress during daily activities.^29^ By improving emotional regulation, mindfulness may help patients experience better daily functioning despite persistent objective motor symptoms. This emotional regulation may also enhance the neuroplastic changes triggered by CMDT, collectively supporting greater functional adaptation.^30^

Postural stability, measured by COPDA, improved significantly in the MC and CMDT groups. As a device-based measurement, COPDA provides supplementary quantitative information beyond what clinical scales can capture. Although UPDRS-III assesses overall motor symptom severity, COPDA specifically supplemented with more sensitive measure of motor function.^75^ No improvement was observed in the AT group, likely because simple-seated aerobic cycling cannot sufficiently challenge postural control system. CMDT trained participants to simultaneously perform dynamic weight shifting, visuomotor coordination, and reactive balance responses during game-based tasks. These tasks require continuous recalibration of centroid positions while performing real-time cognitive executions. Such dual-task conditions activate cortical-subcortical pathways, including the parietal cortex, cerebellum, supplementary motor area, and frontoparietal attention networks, supporting compensatory mechanisms for basal ganglia dysfunction in PD.^76^ By repeatedly training cognitive resource allocation during postural demands, CMDT addresses a core deficit in PD-MCI: the deterioration of dual-task balance control, a major contributor to fear of movement.^20^ Mindfulness seems to provide additional benefits, which may explain the most significant improvement observed in the MC group. Mindfulness may improve proprioceptive precision, increase motor awareness, and facilitate the integration of visual, vestibular, and somatosensory signals.^51^ Improvements in sensory weighting and attentional anchoring may promote more effective postural strategies, reduce unnecessary co-contractions, and enable smoother corrective adjustments. In addition, mindfulness can also reduce anxiety-driven hypervigilance and maladaptive stiffness, both of which can worsen balance performance in PD.^62^

The multidimensional effects observed in the MC group suggest that PD-MCI rehabilitation may benefit from approaches that simultaneously target cognitive, motor, and neuropsychiatric domains rather than relying on a single modality. This pattern is consistent with the concept of experience-dependent neuroplasticity, where the combination of cognitive engagement and task-specific motor practice may facilitate adaptive neural changes, as highlighted in previous work on neuroplastic mechanisms in PD.^77^ The greater improvements observed with the combined intervention also highlight the role of attentional resource limitations in PD-MCI. The addition of mindfulness may have enhanced top-down attentional control and reduced the processing of task-irrelevant information, thereby allowing more efficient management of cognitive-motor demands and reducing dual-task interference.^78^^,^^79^ In parallel, the concurrent improvements across emotional, cognitive, and motor domains support a broader mind-body interaction perspective, suggesting that mindfulness-related changes in emotional regulation and bodily awareness may extend beyond psychological outcomes to influence functional performance.^80^ Taken together, these findings suggest that integrating mindfulness with CMDT may provide a synergistic approach to addressing the multifactorial impairments in PD-MCI.

### Limitations of the study

This study has several limitations that should be considered. The sample size was modest and drawn from a single center, and the 4-week intervention period may not fully capture long-term or delayed treatment effects. The lack of neuroimaging or electrophysiological measures also limits the ability to directly verify the proposed neural mechanisms. Future studies with larger samples, longer follow-up periods, and neurophysiological evaluations are needed to clarify the persistence and neural basis of these effects.

## Resource availability

### Lead contact

Requests for further information and resources should be directed to and will be fulfilled by the lead contact, Jing-Zhi Zhang (170787717@qq.com).

### Materials availability

This study did not generate new unique reagents.

### Data and code availability

• The datasets have been deposited at Mendeley data repository and are publicly available as of the date of publication at [https://doi.org/10.17632/6twwt4rkfk.1].
• This paper does not report original code.
• Any additional information required to reanalyze the data reported in this paper is available from the lead contact upon request.

## Acknowledgments

This study was funded by the Sichuan Province 10.13039/100016694Central Leading Local Science and Technology Development Special Project of China, 2022ZYD0089.

## Author contributions

Conceptualization, R.W. and J.-Z.Z.; methodology, R.W. and J.-Z.Z.; writing – original draft, R.W.; writing – review and editing and supervision, J.-Z.Z.; analysis, S.-M.X.; allocation, B.-x.H.; recruitment, J.C.

## Declaration of interests

The authors declare no competing interests.

## STAR★Methods

### Key resources table

**Table:** 

REAGENT or RESOURCE	SOURCE	IDENTIFIER
**Biological samples**		

Patients with Parkinson’s disease with mild cognitive impairment	Nanjing Mingzhou Rehabilitation Hospital	Clinical participants enrolled in this study

**Deposited data**		

Study dataset	Mendeley Data repository	https://doi.org/10.17632/6twwt4rkfk.1

**Software and algorithms**		

SPSS Statistics version 21.0	IBM	https://www.ibm.com/cn-zh/products/spss
G∗Power version 3.1.9.7	Heinrich Heine University Düsseldorf	https://www.psychologie.hhu.de/arbeitsgruppen/allgemeine-psychologie-und-arbeitspsychologie/gpower

**Other**		

EasyTech Libra proprioceptive balance training system	EasyTech, Italy	https://www.easytechitalia.com/

### Experimental model and study participant details

#### Human participants

Participants were recruited from patients with Parkinson Disease admitted for clinical management between April and July 2025. All participants met the diagnostic criteria for idiopathic Parkinson Disease and mild cognitive impairment in Parkinson Disease according to Movement Disorder Society (MDS) criteria. A total of 53 participants completed the study, including 33 males and 20 females, aged 58–79 years. All participants were Chinese. No significant differences in age or sex distribution were observed among the three groups at baseline. Written informed consent was obtained from all participants in accordance with the Declaration of Helsinki. The study was approved by the medical ethics management committee of Nanjing Mingzhou Rehabilitation Hospital (Approval No. NJKF202503001).

### Method details

#### Study design

This study was designed as a single-blind randomized controlled trial following the CONSORT reporting guidelines. The study was conducted at Nanjing Mingzhou Rehabilitation Hospital between April and July 2025. Participants were randomly assigned to one of three groups: mindfulness plus cognitive-motor dual-task training (MC group), health education plus cognitive-motor dual-task training (CMDT group), or health education plus aerobic training (AT group).

#### Criteria for inclusion and exclusion

Inclusion criteria were as follows: diagnosed of idiopathic PD according to the clinical diagnostic criteria of MDS; meeting MDS Level I criteria for PD-MCI; Montreal Cognitive Assessment (MoCA) score <26; Mini-Mental State Examination (MMSE) score ≥24; age ≥55 years; stable dopaminergic and/or cognition-related medication for ≥4 weeks prior before baseline and unchanged during the study.

Exclusion criteria were as follows: current or past regular mindfulness parctice; use of prescribed cognitive interventions or neuromodulation; history of acquired or traumatic brain injury; recent use of medications known to cause unstable or acute cognitive effects; illicit drug or alcohol abuse within the past five years; other neurological disorders affecting cognition; major psychiatric illness; severe visual, auditory, language, or motor impairments; any medical condition judged by investigators to substantially interfere with cognition, safety, or study adherence.

#### Interventions

All groups received five 60-min intervention sessions per week for 4 weeks. Each session consisted of two 30-min components. The MC group received mindfulness practice combined with cognitive-motor dual-task training (CMDT). The CMDT group received health education combined with CMDT. The AT group received health education combined with aerobic training. All participants received usual care during hospitalization, including medication management and support services, but no specific structured cognitive or motor rehabilitation programs.

#### Randomization and blinding

External personnel prepared numbered cards, placing them in sealed and opaque envelopes. After enrollment, each participant selected an envelope. A researcher not involved in assessment viewed the numbers and assigned participants to one of the three groups. Participants were instructed not to discuss their intervention with other participants or assessors. Outcome assessor and statistical analyst were blinded to both intervention and allocation.

#### Sample size estimation

Sample size was calculated using G∗power 3.1.9.7 software based on a repeated measures analysis of variance (within-between interaction) design. The effect size f was calculated to be 0.45 based on a previous randomized controlled trial that reported significant group × time interaction effects on MoCA scores. To avoid overestimation and ensure a conservative calculation, a medium effect size (f = 0.25) was used in the final analysis. The calculation assumed three groups and two measurement time points. The significance level (α) was set at 0.05 and statistical power (1−β) at 0.80. The correlation among repeated measures was assumed to be 0.5, and the nonsphericity correction ε was set to 1. The required total sample size was calculated to be 42. Considering potential dropout, a total of 60 participants were recruited.

### Quantification and statistical analysis

Statistical analyses were performed using SPSS Statistics version 21.0. Quantitative data are presented as mean ± standard deviation (SD). The biological unit (n) represents individual participants. The Shapiro-Wilk test was used to examine the normality of quantitative variables. Since all variables followed a normal distribution, a two-way repeated-measures analysis of variance was performed to examine the effects of group, time, and their interaction on all outcome measures. The primary effect of interest was the group × time interaction. When a significant effect was identified, post-hoc pairwise comparisonswere conducted. The resulting *p* values were adjusted using the false discovery rate (FDR) correction based on the Benjamini-Hochberg procedure. Categorical variables (e.g., gender) were analyzed using chi-square tests for baseline comparisons. An intention-to-treat (ITT) analysis was additionally performed as a sensitivity analysis using the last observation carried forward (LOCF) method to handle missing post-intervention data. The primary analysis was conducted based on the per-protocol population. All significance tests were two-tailed, with statistical significance set at *p* values < 0.05. Detailed statistical results, including F values, degrees of freedom, effect sizes, and exact *p* values, are reported in the Results section, Tables 1, 2, and 3, and Figure 2. In all figure legends, the symbol # indicates a significant within-group difference (pre vs. post, *p* < 0.05) and the symbol ∗ indicates a significant between-group difference at post-intervention (*p* < 0.05), both derived from post-hoc pairwise comparisons following the two-way repeated-measures analysis of variance.

### Additional resources

Clinical trial registration: This study was registered at ClinicalTrials.gov under identifier NCT06930742. Description: https://clinicaltrials.gov/study/NCT06930742.
